# Occupational therapy graduates' perceptions of their work readiness over their first year of work

**DOI:** 10.1111/1440-1630.70064

**Published:** 2026-01-14

**Authors:** Sarah Miles, Jennie Brentnall, Merrolee Penman, Jo Longman, Gillian Nisbet

**Affiliations:** ^1^ School of Health Sciences, Faculty of Medicine and Health The University of Sydney Sydney New South Wales Australia; ^2^ Curtin School of Allied Health, Faculty of Health Sciences Curtin University Bentley Western Australia Australia; ^3^ The University Centre for Rural Health The University of Sydney Lismore New South Wales Australia

**Keywords:** clinical education, curriculum, framework analysis, new graduates, occupational therapy, practice education, qualitative research, work readiness

## Abstract

**Introduction:**

Employers expect occupational therapy graduates to be ready to work in a broad range of roles, settings, and work contexts. Expected ‘work readiness’ extends beyond discipline‐specific skills and includes the attitudes and attributes essential for success in the workplace. This qualitative research study explored the understanding of work readiness among new graduate occupational therapists in public, private, hospital, and community settings. Further, this study extended on prior research by interviewing participants regarding their perceptions of work readiness over time and the impact of their final placement on their work readiness.

**Methods:**

The participants were six occupational therapy graduates who had completed their final placement in an Australian University Department of Rural Health. This qualitative study, underpinned by pragmatism, used the critical incident technique with two interviews undertaken across the first year of employment. Framework analysis methodology was used to systematically analyse the data inductively and deductively.

**Consumer and Community Involvement:**

None.

**Findings:**

The participants' descriptions were consistent with the work readiness elements of social intelligence, organisational acumen, work competence, and personal characteristics. Overall, three themes for occupational therapy graduates' work readiness were identified: work readiness over time, the importance of relationships across all aspects of work, and the ability to work autonomously. The participants noted that the learning opportunities during a student‐led placement developed their caseload management skills, fostered autonomy and interprofessional working, and enhanced self‐reflection.

**Conclusion:**

The participants in this study highlighted many similar and some new aspects of work readiness, perhaps because of the broad nature of occupational therapy work. As the first study to consider perceptions of work readiness over time, this research identified that graduates may emphasise different work readiness components at different points in their first year of employment. These findings and the graduates' attributions of work readiness to the placement model have implications for developing and supporting graduates' work readiness.

## Plain Language Summary

This study looked at how new occupational therapists felt when they started work. Six graduates did their final placement in a rural area. They were interviewed twice in their first year of work. The study found that being ready for work means having good people skills and being confident with tasks. It showed that readiness changes over time. It also found that relationships and working independently are both important. Graduates said their placement helped them do their job, talk with others, and think about what they learned. It also shows that placement can shape how prepared graduates feel.

**Key Points for Occupational Therapy**

- Occupational therapy graduates perceive working autonomously, building strong relationships, and self‐reflection as important to work readiness.
- Graduates' perceptions of work readiness change over their first year of practice.
- Graduates perceive features of student‐led placements that enhance their work readiness.

## 1 INTRODUCTION

Employers expect graduates to be ready to work across a broad range of roles, settings, and work contexts. Universities are, therefore, expected to prepare graduates to be ‘work ready’ with the skills, attitudes, and attributes that will enable them to succeed in the workforce (Peersia et al., 2024). The current work readiness literature in health primarily focusses on medically oriented health professions such as nursing, medicine, pharmacy, and dentistry (Orr et al., 2023). How work readiness presents in disciplines spanning the broader health and social care sectors, including allied health professions such as occupational therapy, has received less research attention. Furthermore, the predominance of cross‐sectional research presents work readiness as a static concept, without considering how it may evolve over time (Orr et al., 2023; Walker & Campbell, 2013). Understanding the concept of work readiness and its evolution over time will enable universities and workplaces to better prepare and support graduates across the diverse health‐care landscape.

Work readiness is defined as “the extent to which graduates are perceived to possess the skills, attitudes, and attributes that render them prepared or ready for success in the workplace” (Caballero et al., 2010, p. 42). It extends beyond discipline‐specific skills, encompassing the attitudes and attributes necessary for success in the workplace (Caballero & Walker, 2010; Jackson, 2019). In their seminal Australian study with participants from engineering, science, commerce, business, accounting, law and finance occupations, Caballero et al. (2010) identified four domains of work readiness: social intelligence, organisational acumen, work competence, and personal characteristics. Social intelligence includes the skills and attributes needed to communicate with various people, work within a team, manage interpersonal conflict and seek support. Organisational acumen includes department knowledge, organisational policy and procedures knowledge, maturity, and professional development/lifelong learning. Work competence includes clinical skills, technical knowledge, experience, confidence, and responsibility. Personal characteristics include self‐awareness, adaptability, flexibility, resilience, and stress management (Caballero & Walker, 2010; Walker & Campbell, 2013). These four domains have since been validated with descriptors adapted for nursing and medical graduates (Syed Aznal et al., 2019; Walker et al., 2015, 2013). This four‐domain structure has then been applied to the Australian allied health disciplines to develop an allied health work readiness scale (Lawton et al., 2022). However, this has mainly included physiotherapy, with limited representation of 11 other allied health professions. A newer study proposes an additional two domains to the allied health work readiness scale (profession‐specific knowledge and skills and professionally relevant experiences) (Lawton et al., 2025).

Research into the work readiness of health professional graduates has largely been conducted within the nursing and medical professions. Research highlights consistent gaps in the personal characteristics and organisation acumen domains. Both managers and graduates have identified difficulties in personal attributes such as resilience, flexibility, conflict resolution and stress management skills, and difficulties in the organisational domain, including caseload and time management, administrative tasks, and understanding health system processes and funding structures (Merga, 2016; Walker et al., 2024, 2013). Graduates also reported limited transferable skills, particularly when their student placements do not align with future workplaces (Merga, 2016). A more recent qualitative study of specific to allied health identified the importance of clinical reasoning, communication, collaboration, technology skills, and professionalism in being work ready (Walker et al., 2024). They noted persistent challenges in critical reflection, managing expectations, conflict resolution skills, professionalism, understanding organisational processes and funding systems, and transitioning from university to the workplace (Walker et al., 2024).

Much of the existing research has focussed on defining and validating work readiness through quantitative measures, often using single interviews or scales, without exploring how work readiness may evolve (Lawton et al., 2022; Syed Aznal et al., 2019; Walker et al., 2015). These studies have been primarily conducted within the public health sector, with occupational therapy representing only a small proportion of the sample and typically discussed within a broader context of allied health (Lawton et al., 2022; Merga, 2016; Walker et al., 2024). As a result, specific findings and applications to the occupational therapy profession and its diverse workplace settings remain unclear. A more nuanced understanding of work readiness, incorporating consideration of whether it is a static or evolving concept, is needed to support occupational therapy across diverse practice settings.

Many of the findings about work readiness in broader health research align with the existing literature in occupational therapy regarding the preparedness of graduates for practice. The occupational therapy literature on graduate preparedness takes a more practical approach of identifying competencies, knowledge, and skills for practice, rather than graduates' overall attributes and attitudes reflecting work readiness (Plastow & Bester, 2020). None‐the‐less, from this literature, it could be inferred that occupational therapy graduates have perceived challenges with some components of work readiness. In particular, interacting with other health professionals and navigating workplace cultures (Moir et al., 2021), which is considered part of the social intelligence domain of work readiness; difficulties in applying evidence‐based practice (Gray et al., 2012; Murray et al., 2020), working with complex cases (Murray et al., 2020; Naidoo et al., 2014), and managing and prioritising a caseload (Adam et al., 2013; Murray et al., 2020), which are relevant to the work competence domain; and difficulties in developing professional self‐confidence and role identification (Hodgetts et al., 2007) and in navigating workplace cultures (Moir et al., 2021), which fall within the organisational acumen domain. Much of the preparedness for practice literature identifies the significance of the placement experience in fostering these competencies, knowledge, and skills. Still, it is unknown how placements may foster attitudes and attributes of work readiness within occupational therapy (Plastow & Bester, 2020).

Contemporary occupational therapy placement models are reported to have certain characteristics that may be more likely to foster work readiness. These placement models aim to use different aspects of experiential learning to fill these gaps and better prepare students for the dynamic health‐care environment (Kay et al., 2019; Nyoni et al., 2021). Specifically, peer learning during placements is reported to enhance teamwork, autonomy, reflection, feedback skills, conflict management, and support‐seeking abilities (Beveridge & Pentland, 2020; Dancza et al., 2013; Sevenhuysen et al., 2017). Long‐arm supervision, in which an experienced supervisor is located remotely from the student for most of the time, aims to foster independence, time management, self‐management, and lifelong learning (Clarke et al., 2014; Lau & Ravenek, 2019). Professional identity may be developed through role‐emerging placements, where students are placed in sites without an established occupational therapy programme or role (Clarke et al., 2014; Lau & Ravenek, 2019). Interprofessional collaboration, professional reasoning, and evidence‐based practice can be further developed through student‐led and service‐learning placements, where students are responsible, with appropriate support, for delivering a health‐care service (Beveridge & Pentland, 2020; Dhunpath et al., 2019; Lising et al., 2025). Overall, these contemporary placement models are increasingly popular in allied health curricula; however, many studies evaluating these models lack empirical rigour and long‐term follow‐up (Beveridge & Pentland, 2020; Nyoni et al., 2021). This limits understanding of how placement features may specifically contribute to their effectiveness, and quality in preparing students to be work ready needs to be explored further beyond immediate post‐placement surveys (Nyoni et al., 2021).

In summary, being work ready is essential for graduates to succeed in the workforce, although preparedness for practice is more often the focus in the occupational therapy literature. Exploring the perspectives of work readiness and the influence of placements on this, among new graduate occupational therapy graduates, will provide valuable insights in the development of supportive strategies by universities and workplaces. Additionally, understanding the evolution of graduates' perceived work readiness over time will have direct implications for workplaces and preparing graduates for the diverse health‐care landscape. This study investigated two research questions:

1. What do new graduates understand work readiness to be?
2. How do graduates perceive that features of their final placement contributed to their work readiness?

## 2 METHODS

### 2.1 Study design

This exploratory study adopted a pragmatist paradigm (Biesta, 2010; Morgan, 2014) to investigate the perceptions of occupational therapy graduates regarding what it means for them to be work ready. Qualitative interviews using the critical incident technique (Lister & Crisp, 2007) were used to identify graduates' workplace experiences. Framework analysis was employed to systematically interpret key themes and patterns related to occupational therapy graduates' work readiness, and to examine how features of their final placement influenced this readiness (Gale et al., 2013). The University of Sydney Human Research Ethics Committee approved this study (Project number: 2018/351).

### 2.2 Study context and recruitment

All participants had completed their final placement through a University Department of Rural Health (UDRH) in Australia. UDRH is funded through the Australian Federal Government to support allied health placements in rural and remote communities (Department of Health, 2019). By fostering interprofessional learning and immersive rural experiences, the programme aims to enhance graduates' readiness for practice, improve health outcomes for underserved communities, and encourage long‐term rural employment (Department of Health, 2019).

Thirty‐six pre‐registration occupational therapy programme graduates who completed their final, 8‐ to 10‐week‐long occupational therapy placement at the UDRH in 2017 or 2018 were invited to participate in this study. All were graduates from three Eastern Australian universities' pre‐registration occupational therapy programmes. The UDRH's administration team emailed research invitations to the graduates approximately 4 months after their programme completion. Participation in this study was voluntary, and graduates were recruited to the study only if they replied to the email consenting.

### 2.3 Data collection

The lead author (SM) conducted semi‐structured interviews with each participant approximately 3 months after they commenced employment and again 6 months later. Using critical incident technique (Lister & Crisp, 2007), graduates were asked about commonplace significant events in their average week and examples of the skills and attributes they utilised throughout their work days. They were asked to provide examples of where they had and had not felt work ready. This elicited detailed narratives from participants about their understanding of work readiness and how they perceived it related to their current working environment (Halcro & Roberts, 2024). After discussing their current work and work readiness, which built rapport and understanding between the participants and interviewer, the participants were then asked which features of their placement supported or did not support their work readiness. See Data S1 for interview questions.

### 2.4 Data analysis

Framework analysis method (Gale et al., 2013; Goldsmith, 2021) was used to identify key themes and patterns regarding occupational therapy graduates' work readiness from across the interviews, key literature, and Caballero et al.'s (2010) work readiness framework (Gale et al., 2013). The framework analysis method involves a series of distinct stages: data familiarisation, developing an analytical framework, applying the framework to the data, charting and indexing the data, and mapping and interpreting the results (Gale et al., 2013). All coding was completed within NVivo 14 (Lumivero, 2023).

#### 2.4.1 Data familiarisation

SM, MP, and JB read the first interview transcript to familiarise themselves with the content. Each author coded the first paragraph of the transcript using line‐by‐line coding and compared and discussed differences in interpretation. As SM was also an educator for four participants, this joint coding helped the researchers focus on the data and decreased potential biases by facilitating coding on a conceptual level to ensure relevance (Holton, 2007). The first paragraph was then discussed as a team (SM, MP, and JB), and common codes were agreed upon. The rest of the interview was then coded individually by these team members, and additional inconsistencies were discussed to refine common codes. Only occupational therapist team members were present during this stage. To support reflexivity, the interview was then coded by an additional team member who was not an occupational therapist (JL), and further organisation and interpretation of the data was undertaken to establish the initial coding framework. Using the initial codes, two further transcripts were independently coded by two researchers (SM and MP). Cohen's Kappa coefficient query was used to calculate the agreement between coders, accounting for chance agreement, for each code in each of these two transcripts (McHugh, 2012). Within each transcript, codes with Kappa agreement coefficients of less than 0.75 were checked, discussed, and resolved by the team.

#### 2.4.2 Developing and applying an analytical framework

After coding the first three of the 12 transcripts, SM reviewed the codes and their relationship to the research question and the literature (Gale et al., 2013). Many of the 25 codes inductively generated aligned with Caballero et al.'s (2010) four overarching domains. The framework analysis process involves the development of ‘categories’ (Gale et al., 2013); however, for consistency and clarity within this article, categories are referred to as ‘domains’. Therefore, the four overarching domains of social intelligence, organisational acumen, work competence, and personal characteristics served as the basis for this study's analytical framework (Gale et al., 2013). Table 1 provides an overview of the analytical framework, presenting the codes for each domain.

**TABLE 1:** Analytical framework.

Domain	Codes
Domain 1: Social intelligence	Communication Conflict resolution/managing interpersonal conflict Healthy boundaries within the workplace^a^ Relationship building or interpersonal orientation Relationships with staff and team, including seeking support from others Working within a team Increased responsibility^a^
Domain 2: Organisational acumen	Adapt to a working environment and work routine^a^ Knowledge of funding systems and organisational systems Lifelong learning^a^ Professional identity and role differentiation^a^ Professionalism^a^ Understanding of practice limitations^a^
Domain 3: Work competence	Administration tasks^a^ Caseload management and prioritisation^a^ Clinical reasoning and confidence in skills Knowledge of technical skills and theories Organisational and time management skills
Domain 4: Personal characteristics	Confidence Ability to be autonomous and work independently^a^ Flexibility Initiative Positive attitude^a^ Resilience Stress management

^a^ Codes unique to this study.

An additional code, which could overlap with any of the codes in Table 1, was retained to identify all placement‐related comments that might address Research Question 2. SM then used the analytical framework to code each transcript. MP checked all coding to ensure consistency, and any discrepancies were discussed with the team.

#### 2.4.3 Charting the data into the framework matrix

Once all the data had been coded using the framework, the data were summarised using one matrix for each of the four domains and an additional matrix for placement data (Gale et al., 2013). Each matrix comprised one row per participant and one column per code. Two additional rows in each matrix summarised all the data for the first and second interviews, respectively (one column per code). The researchers charted and summarised two domains (organisational acumen and work competence) to ensure a consistent data interpretation approach. SM then summarised the rest of the data for discussion with the team. For an illustration of this process, see Data S2.

#### 2.4.4 Interpreting the data to derive themes

During the interpretation stage (Gale et al., 2013), and to answer Research Question 1, the researchers sought to make sense of the data, drawing connections between the matrices for each work readiness domain and the participants' placement sites and current workplace settings. The researchers discussed and refined potential overall themes to represent common topics within and between the matrices. In this stage, the data were also examined across two time points to see how themes developed or changed over the participants' first year of employment.

Finally, to address Research Question 2, the participants' perspectives on the role of placement features in their subsequent work readiness within the four matrices and within the placement matrix were interpreted. Statements relating to the participants' final placement at the UDRH were mapped to features of the placement instituted by design (see Table 2) or incidentally. Features of or comparisons to the participants' prior placements were also noted.

**TABLE 2:** Placement model design features.

Placement model type	Design features of the UDRH final placement
Role emerging (Clarke et al., 2014; Lau & Ravenek, 2019)	No employed occupational therapist at the placement site Placements occur in pre‐schools, schools and aged care homes
Student‐led/service‐learning (Beveridge & Pentland, 2020; Dhunpath et al., 2019; Lising et al., 2025)	Placement sites are located within underserved communities. Placement activities are co‐designed with placement sites and UDRH supervisor to match student interest areas, learning outcomes, and site needs With appropriate support and scaffolding from the supervisor, students lead all aspects of service delivery and manage their workloads Students develop, manage, and implement services such as individualised and group interventions, whole‐class activities (if school/pre‐school placement), site initiatives, staff education, and quality improvement projects Students organise the modality of supervision, such as on‐site visit times, phone calls, and meetings with core staff on site
Long‐arm supervision (Clarke et al., 2014; Lau & Ravenek, 2019)	Provided by a UDRH‐employed occupational therapist Located remotely from the student Face‐to‐face approximately two half‐days per week Remote modalities including phone calls, emails, text messages, review of written student documentation, review of peer and self‐reflections, online student debriefs, tutorials, and clinical case discussions. Flexible depending on student need, placement timing, and supervisor availability.
Peer learning (Beveridge & Pentland, 2020; Dancza et al., 2013; Sevenhuysen et al., 2017)	Paired student allocations to each clinical site Weekly peer reflection and debriefs (informal and formal) Encouragement to share resources across sites and students Access to previous student‐developed resources Clinical note audits of previous students' notes to develop documentation skills (and learn about the clients)
Interprofessional learning (Patel et al., 2025)	Where possible, co‐allocation with student pairs from other disciplines Joint sessions and therapy plan with students from other disciplines Weekly multi‐disciplinary education day

## 3 POSITIONALITY STATEMENT

This study was led by a PhD candidate (SM) working in health education, with professional experience in coordinating student placements and mentoring new graduates. SM was directly involved in the development of the placement model under investigation and in supervising students who participated in the placements. This dual role as both practitioner and researcher offered valuable contextual insight and also required ongoing reflexivity to navigate potential biases and power dynamics. The use of the critical incident technique and the order of interview questions were chosen within the study design as one way to navigate this.

The rest of the research team comprised five members with diverse yet complementary expertise. Three were experienced academics with substantial backgrounds in work‐integrated learning (MP, JB, and GN), including two registered occupational therapists (MP and JB), whose clinical and educational perspectives informed the interpretation of student experiences. The fourth team member (JL) was a qualitative research specialist who provided methodological guidance and supported the integrity of the analytical process. Reflexivity was embedded throughout the research process through regular team discussions. These strategies enabled the team to critically reflect on their assumptions, positionalities, and interpretations, thereby enhancing the trustworthiness and depth of the study.

## 4 FINDINGS

Six participants took part, each completing two phone interviews lasting 30 to 40 min. All participants had graduated from one university (of the three universities that were approached). While each participant was with the same employer at the time of both interviews, two participants had transitioned to a different ward, and one had commenced an additional weekend position in an aged care home. All participants were working in metropolitan areas, with two participants having moved cities to gain employment, attributing completing their rural placement with giving them the confidence to move. See Table 3 for participant demographics.

**TABLE 3:** Participant demographics.

Characteristic	Number of participants
Course	
Undergraduate	3
Graduate entry masters	3
Gender	
Male	1
Female	5
Placement site	
Aged care home	2
Pre‐school/school	4
Graduate workplace (primary)	
Residential aged care home	2
Inpatient hospital	2
Paediatric private practice	1
Community mental health	1

### 4.1 OT graduates' work readiness according to the analytical framework

The participant's descriptions of work readiness were found to be broadly consistent with one or more of the four overarching domains of work readiness (social intelligence, organisational acumen, work competence, and personal characteristics; Caballero et al., 2010; Walker et al., 2013). However, some codes that expanded on the original inclusions described in the literature were added to fully encompass the data within this study (Table 1). The *social intelligence* domain was expanded to include the importance of establishing healthy boundaries within the workplace and the increased responsibility of being a graduate compared to being a university student. The *organisational acumen* domain was broadened to encompass the need for establishing and developing a professional identity and role differentiation, emphasising the importance of professionalism and understanding practice limitations. Lifelong learning was also added, which participants noted was underpinned by the ability to self‐reflect. The *work competence* domain was expanded to include handling administrative tasks and developing caseload management and prioritisation skills, which participants noted were crucial for completing all required tasks in a day. Finally, the *personal characteristics* domain was expanded to include the ability to take initiative and maintain a positive attitude, which participants associated with working independently and autonomously.

### 4.2 Theme 1: work readiness emphasis over time

When comparing participants' data across the two interviews, it became evident that graduates highlighted different aspects of work readiness over time. The participants in their earlier interviews emphasised the process and practicalities of transitioning from university to work. They emphasised the importance of *‘understanding the health care system’* [Participant 4, Interview 1 (P4–1)], including the environments in which they worked (funding and organisational), as well as their professional identity (organisational acumen) *‘how I'm different to a PT’* (P1–1) and the clinical skills required to perform the role such as *‘assessment skills, clinical reasoning skills (P3‐1) and quickly developing a session plan independently’ (P5–1)* (work competence). Later in the graduate year, the participants acknowledged that they still had a lot to learn. Their emphasis shifted to the personal characteristics of work readiness, such as self‐reflection and independent problem‐solving, as illustrated by this quote *‘what went well, what didn't go well, what can I do to improve that next time?’* (P3–2) to overcome knowledge gaps. The participants identified the ability to reflect and independently problem‐solve as the tools that enabled them to identify further development of work readiness over time.

During the earlier interview, many participants discussed the challenges of working full‐time and their lifestyle changes. Some noted life skills needed, such as getting to work on time (organisational acumen), *‘transitioning from a uni student lifestyle’*, and ‘*manag[ing] a longer day's work’* (P3–1). Other participants noted the change in mindset from a student *‘told to take every opportunity’* to a clinician *‘learning to only spread myself as far as I can’* (P4–1), acknowledging *‘there's a lot of responsibility’* (P2–1) (social acumen). During the later interview, the participants shifted emphasis to the personal characteristics required to succeed within the workplace, including that *‘resilience … builds with experience’* (P3–2) and *‘learning to separate myself from my work’* (P4–2).

During the earlier interview, the participants discussed being unprepared for the complexity and variety of workplaces (organisational acumen), acknowledging that although they were aware of this from their studies, *‘it's not the same’* (P4–1). Commenting that navigating these complex systems (organisational acumen) was ‘*tricky’* (P6–1), time was required to understand them. However, during the later interview, although the participants discussed funding systems as challenging to navigate (organisational acumen), they acknowledged that they would need to seek support as required, *‘I go to my team’* (P3–2) (social intelligence and organisational acumen).

Similarly, during the earlier interview, the participants emphasised the importance of ‘*knowledge of the field itself… paediatrics, mental health, etc’* and the importance of ‘*understanding recurring interventions, assessments in the field’* (P4–1), across all areas of practice (work competence). They discussed the skills and clinical reasoning needed to implement and understand the therapy process as a core part of their day, with the ability to *‘develop a session plan quickly and independently’* (P5–1) being crucial to success in their job. However, many participants reported decreased confidence in their clinical skills, commenting they ‘second‐guess’ (P1–1; work competence). As participants progressed through their graduate year, they reflected on ‘*accepting the fact.. you don't know everything’* (P5–2) at the end of their university degrees. They acknowledged that many skills would be learnt on the job, discussing the importance of having realistic expectations and the strategy of *‘reflecting upon… clinical skills… and reasoning’* (P3–2). They also described the importance of understanding their professional identity and the role of an occupational therapist in the setting they worked in (organisational acumen). This was especially evident in the generalist roles in the aged care and mental health sectors with the need to *‘understand what we do and how that's different’* (P1–1) when multiple professions could undertake the role. In the later interview, the participants expanded this to include the need to explain or advocate for the occupational therapy profession as a vital work‐ready skill. This was reflected in their examples of other professions not understanding their scope such that, *‘you need to stand up for OT … the consultant doesn't fully understand the role of OT’* (P3–2).

### 4.3 Theme 2: relationship building

The participants identified relationship‐building as central to developing all areas of work readiness. Relationship building was considered essential for developing a therapeutic relationship, achieving therapeutic outcomes, understanding team members and their roles, collaborating within a team, and seeking assistance from the team or workplace supervisor when needed. Building strong relationships (social intelligence) was instrumental in developing organisational acumen, work competence, and personal characteristics.

All participants discussed the importance of strong relationships to achieve quality patient and therapy outcomes (social intelligence). During the first interview, the participants identified relationship‐building skills such as *‘being empathetic’ and ‘active listening skills’* (P5–1). During the later interview, the participants focussed on the teamwork component of relationship building for patient quality outcomes. The participants discussed the importance of advocating and *‘negotiat (ing) with colleagues’* to *‘prioritise what are we doing … and who is best’* (P6–2) within the team to complete the service (social intelligence). Strong relationships within the team were therefore recognised as a core component in enhancing patient outcomes (social intelligence): *‘You need to… work in a team cohesively… be on the same page’* (P5–2).

The participants felt that deep relationships with the team or work supervisor meant they could access support when they lacked the required skills or knowledge (social intelligence). *‘If I'm not sure… go to a senior occupational therapist or my supervisor … talk through what I was thinking’* (P2–1).

### 4.4 Theme 3: working autonomously

The participants commonly listed working autonomously as one of the top skills in their definition of work readiness. The level of importance participants attributed to this did not change between interviews; however, they spoke about it less frequently in the second interview, while still emphasising its significance when it was mentioned.

The participants focussed on the importance of *‘working independently’* and *‘making … clinical judgement(s) independently’* (P4–1). This was often discussed, along with *‘flexibility… to change with the plans’* (P3–1) and willingness to *‘just have a go’* (P6–1). The participants also discussed the importance of *‘a positive attitude’, ‘working hard’, ‘learning’, ‘being ready to jump in’, and ‘doing things properly’* (P4–1). They identified the ability to manage and complete documentation and administrative tasks autonomously as essential for workplace success: *‘That's a huge part to being work‐ready, being able to manage all the documentation’* (P5–1). They also outlined the importance of managing large caseloads and advocating for a suitable caseload as necessary, acknowledging this as an area where they felt unprepared. *‘My caseload … I cannot handle any more right now, and I've made that clear’* (P4–1). The participants also linked this to stress management strategies and how this may impact their ability to complete their roles effectively (personal characteristics).

### 4.5 The contribution of placement features to graduate work readiness

The participants consistently identified three key design elements within the placement as helpful in building their work readiness: long‐arm supervision, interprofessional learning opportunities, and working in pairs. Additionally, all participants linked the placement context of travelling to a rural setting and the experience of working with diverse populations to increasing their confidence in transitioning as a new graduate.

All participants acknowledged that long‐arm supervision helped develop their ability to work autonomously (personal characteristics), as reflected when describing their placement supervision. *‘You figure out what you don't know … seek supervision rather than being shown and being told.’* (P4–2) They described their increased ownership of the service delivery as helpful in developing critical thinking. For example, one participant reflected on previous placements: *‘You did the same things, so there wasn't the same critical thinking and problem solving that we experienced’* (P4–2) (social intelligence and work competence). The participants identified that not having a supervisor on‐site prepared them to advocate for and utilise supervision well (social intelligence), such as ‘scheduling regular debriefs … discussing concerns and hesitations’ (P6–2), describing it as *‘similar in my current workplace’* (P6–2). The participants also discussed long‐arm supervision as being useful in building skills around organisation, prioritisation, and caseload management (organisational acumen). Additionally, the participants identified that the combination of the long‐arm supervision coupled with weekly reflection tasks gave them more confidence: *‘having that structured, supportive environment made it a lot easier to be able to go into my job … know how to schedule my day … if I have two clients who are really difficult, not to have them back‐to‐back but spread them out’* (P6–2). However, the participants still discussed managing expectations and workloads in their new graduate roles as areas they were not prepared for, as illustrated by this quote: *‘Another aspect is large caseload… I have limited say regarding who should be or should not be on the program. Whereas I was able to determine my own caseload during my placement.’ (P1–1)*.

The participants discussed how interprofessional learning opportunities on placement assisted in understanding the occupational therapy role, the roles of other disciplines, and how different disciplines work collaboratively (social intelligence and organisational acumen). *‘During my placement, I got to work collaboratively with speech therapists… learning how allied health can work together’* (P1–2), adding *“this is so helpful now”* (P3–2). Many participants acknowledged limited opportunities to work collaboratively with other disciplines on real cases in their course programmes prior to placement. An unexpected design feature was the access to interprofessional supervision, which participants identified as a positive factor in developing their knowledge of and the ability to communicate with other disciplines in their current workplaces (social intelligence).

The participants identified that working in pairs and with other students during placement developed their communication and conflict‐management skills (social intelligence). The participants reflected that this helped them develop skills in working with different types of people and independently resolving conflicts (social intelligence): *‘When you're in a traditional placement with a one‐on‐one supervisor, you just go to your supervisor… But here if I had any conflict… I tried to address it then… reflect on it later, so that was a good experience’* (P4–2). However, the participants also reported still feeling anxious and unsure about how to have difficult conversations with clients, families, and team members. Some participants commented that the workplace was the first time they needed to have these challenging conversations and, *‘I had a lot of difficulties liaising with (emotional) family members’* (P2–1).

Additionally, as this placement was situated rurally, all participants discussed leaving ‘home’ for a while as an experience that developed their confidence and how this translated across all areas of work readiness. *‘Having gone rural and done the long‐arm placement, I was a lot more ready to just go out on my own.’* (P6–1). They also identified that *‘being in the country, it was a lot wider groups of people. You had to be a lot more culturally sensitive … and be creative as there isn't the resources available*” (P5–1).

## 5 DISCUSSION

This study investigated the understanding of work readiness among occupational therapy new graduates and their perceptions of how characteristics of their student‐led placement influenced their work readiness. When exploring the work readiness skills, attitudes, and attributes, the findings for occupational therapy graduates were similar to those described by Walker et al. (2013) for medical and nursing graduates, although some additional nuances were included. Three priority themes for occupational therapy graduates' work readiness, which cut across the four overarching work readiness domains (Cabellero & Walker, 2010; Walker & Campbell, 2013), were interpreted: work readiness over time, the importance of developing relationships across all aspects of work, and the ability to work autonomously. Additionally, when examining the design features of the placement model, graduates identified the long‐arm supervision, interprofessional learning opportunities, and working in pairs as key elements in developing their work readiness. The implications of these findings are now discussed in terms of (1) the broad nature of occupational therapy work, (2) changes in work readiness emphasis over time, and (3) the influence of placement on the development of work readiness.

The broad nature of the occupational therapy profession means that graduates work across varied and diverse settings, with a large number working in the private and not‐for‐profit sectors (Occupational Therapy Board, 2022). This may explain the extensions identified in this study to the original Walker et al. (2013) work readiness domains, especially the number of additions to the organisational acumen domain. Graduates' perceptions of their work readiness may also vary because of differences in university coursework, placement learning experiences, and graduate outcomes (Daniels & Brooker, 2014). Successful graduates need to recognise that the work readiness skills, attributes, and attitudes developed throughout their university degree are transferable across different workplace settings (Merga, 2016; Moir et al., 2022).

University curricula must continue to provide students with learning opportunities to reflect on the various aspects of work readiness and how this translates into different workplace settings. The participants in this study identified the ability to work autonomously, particularly in caseload management and critical thinking, as core to their work readiness. They highlighted the opportunity to practice semi‐autonomously during placement as beneficial. With new graduates still reporting difficulty in prioritising, time and caseload management areas (Moir et al., 2021; Nayar et al., 2013; Seah et al., 2011), universities may consider how they implement placement supervision models to develop the skills, attitudes, and attributes required before students graduate. This is particularly relevant with the continued shift to fee‐for‐service programmes such as the National Disability Insurance Scheme (NDIS) and the National Aged Care reforms, where workplaces need graduates to be able to manage a caseload quickly and efficiently. The participants also highlighted the value of interprofessional learning and the benefits of working collaboratively with other professions. This type of learning plays a vital role in enhancing students' teamwork skills, improving patient outcomes—including person‐centred care—and fostering professional identity and confidence (Patel et al., 2025). To support these outcomes, universities may consider incorporating and promoting interprofessional placements, where students can work together on a shared caseload. Additionally, the participants considered strong relationships vital to every aspect of work readiness, so building these relationships may warrant consideration and reinforcement through university curricula, placements, and transition supports (Yu et al., 2021). This may include highlighting the importance and value of informal support, such as hallway and peer conversations (Moir et al., 2022).

Graduates in this study identified and focussed on different work readiness components over time, shifting from a more internally focussed perspective (e.g. practicalities of the job, needing to do things correctly) to a more externally focussed perspective (e.g. advocacy role). This suggests that graduates require a foundational level of work readiness early in their work experiences. However, this study found that different aspects of work readiness may further develop and the emphasis may shift through lived experience. This process or development of work readiness aligns with the work of Murray et al. (2020), describing the learning thresholds for new graduate occupational therapists. Although students and graduates cannot be prepared for everything, this research could inform university curricula (including placements) such as by guiding discussions with students about their work readiness. These discussions should focus on the concept of work readiness, its transferability across different workplace settings, and its changing emphasis over time. It is important to recognise that while graduates often enter the workforce with high expectations, they are still in the process of learning, and confidence typically develops through experience (Moir et al., 2022; Walker et al., 2024). New graduate programmes could better support graduates by recognising and normalising that work readiness may look different for different people, in different workplaces, and at different points in time. These programmes may benefit from having graduates reflect on their current work readiness in the workplace and then develop strategies needed to further their professional growth. Additionally, when considering work readiness through scales and quantitative measures (Peersia et al., 2024), it is essential to factor that the participants may highlight different aspects in their ratings over time. These findings also suggest that research may need to explore whether employers and supervisors also hold different expectations for work readiness at different stages of the graduate year.

Finally, the participants in our study reflected that their student‐led placements supported their work readiness. These placements utilise many concepts and theoretical underpinnings of role emerging, student‐led/service learning, long‐arm supervision, and peer learning placements, which have been discussed extensively in the occupational therapy literature (Beveridge & Pentland, 2020; Kay et al., 2019; Syed & Duncan, 2019). While further rigorous placement outcome research is needed, our study expands and supports the existing literature, indicating that the learning opportunities afforded by these contemporary placement models are perceived as valuable to graduates (Beveridge & Pentland, 2020; Kay et al., 2019; Syed & Duncan, 2019) with a longer term perspective. Our research reinforces the need for occupational therapy curricula (including placements) to develop students' ability to manage a caseload and work autonomously. Students also need to develop the ability to utilise self‐reflection tools, acknowledging that being work ready is challenging and knowing how and when to seek support is vital (Moir et al., 2022). Finally, students require the necessary tools to establish effective relationships across all aspects of their work.

## 6 LIMITATIONS

This study has several limitations. The participants who self‐selected into the study may have been motivated by positive experiences in their UDRH placements. The lead author (SM) was involved in both the design of the placement model and the interview process. This offered a unique insider perspective and important contextual insights, but may also have introduced unconscious bias in data collection, interpretation, and reporting. To mitigate this, the research team engaged in regular reflexive discussions and included members with diverse disciplinary backgrounds and perspectives. This collaborative approach is a strength of the analysis.

Although students from three universities were invited to participate, all participants came from a single university. They represented two distinct entry‐level occupational therapy programmes with differing cohorts and curricula, but their experiences may not reflect those of graduates of other university curricula and placement models.

While the range of practice areas represented was broadly reflective of typical new graduate roles, it did not encompass the full spectrum of occupational therapy practice. The small sample size further limits the breadth of perspectives captured, although the depth of qualitative insights provides a valuable understanding of their experiences.

Finally, while the findings are specific to occupational therapy graduates in Eastern Australia, they may have relevance for supervisors, employers, other disciplines, and international contexts where contemporary placements are used to support workforce readiness.

## 7 CONCLUSION

To our knowledge, this study was the first to examine work readiness exclusively through an occupational therapy lens. Conducting qualitative research across two time points in a new graduate's first year of work provided a unique perspective, indicating that graduates may focus on different work readiness components over time. In the first 6 months, graduates focussed more on internal work readiness factors, moving to more external factors in the second half of their graduate year. Occupational therapy graduates identified the student‐led placement model as valuable in developing their work readiness.

## AUTHOR CONTRIBUTIONS

All authors served as PIs, interpreted the data, and contributed to the study. The first author (S.M.) was a major contributor to writing the manuscript. All authors have read and approved the final manuscript.

## CONFLICT OF INTEREST STATEMENT

The authors have no conflicts of interest to declare.

## DECLARATION OF USE OF ARTIFICIAL INTELLIGENCE

The plain language summary was entered into Co‐Pilot (SM) with the prompt to ‘rewrite to a Flesch Kincaid reading level no greater than 8’. The output was checked, refined, and confirmed by the authors before inclusion.

## Supporting information


**Data S1:** Interview Questions.

**Data S2:** Extract of charting from the *Personal Characteristics* matrix.

## Data Availability

Research data are not shared.
