# Supplementary material for: Occupational therapy graduates' perceptions of their work readiness over their first year of work
Source: Aust Occup Ther J. 2026 Jan 14;73(1):e70064. doi: 10.1111/1440-1630.70064 (PMC12801176; doi:10.1111/1440-1630.70064)
Supplement: Supplementary file 1 — Data S1: Interview Questions. [file AOT-73-0-s002.docx]

Supplementary 1: Interview Questions

Q1

Can you describe your average week/day? Can you give examples of particular skills/attributes that you require throughout the day?

Q2

What do you understand work readiness to be for new OT graduates?

Prompt: What Knowledge, skills, attributes are involved?

Q3

Can you describe an situation at work where you believe you had the work readiness skills/knowledge needed to manage the situation?

Q4

Can you describe an situation at work where you believe you did not have the work readiness skills/knowledge needed to manage the situation?

Q5

Thinking back to your placement at UCRH can you give some examples of where this particular placement supported your work readiness

Prompt: can you think of particular examples of specific work readiness skills that you developed and how that has transferred to your current work place?

Q6

Thinking back to your placement at UCRH can you give some examples of where this particular placement did not support your work readiness

Q7

What could be changed on the UCRH placement to better support your work readiness generally and for your current work setting?
