# Supplementary material for: Occupational therapy graduates' perceptions of their work readiness over their first year of work
Source: Aust Occup Ther J. 2026 Jan 14;73(1):e70064. doi: 10.1111/1440-1630.70064 (PMC12801176; doi:10.1111/1440-1630.70064)
Supplement: Supplementary file 2 — Data S2: Extract of charting from the Personal Characteristics matrix. [file AOT-73-0-s001.docx]

Supplementary File 2: Extract of charting from the *Personal Characteristics* matrix

Table 2 illustrates this with an extract from the personal characteristic matrix. The columns show three of the seven codes, with two rows presenting the overall perceptions and views of Participants 4 and 5, and two rows presenting the combined first or second interviews of all participants. The matrix cells contained summaries of the coded data and/or illustrative participant quotes.

Table 2: Extract of charting from the *Personal Characteristics* matrix

| **Category 2: Personal Characteristics** | | | |
| --- | --- | --- | --- |
|  | Confidence | Ability to be autonomous and work independently | Flexibility |
| Participant 4 | Confidence develops over time, “I think when I just first started, I was doing similar things, but I wasn’t as confident, so I wasn’t taking on as much. However, a year on now I feel like I can do it from the back of my hand, it's just routine now in a way.” Interview 2 | “So I think being able to work independently and making your decisions based on a clinical judgement that you have made is really important.” Interview 2  “I think a second one is probably my ability to work independently without having to consistently consult a member of - a senior clinician or a supervisor.” Interview 2 | Need to be flexible within the day to changing and competing priorities in workloads. |
| Participant 5 | Confidence is needed to talk to other professionals  “It’s scary, especially with consultants who have been in their field for years and years and you are like this new grad who needs to stand up for OT a bit” Interview 2 | Completing a rural placement and moving helped the graduate be more independent as they moved away from home for the first time. | The need to make a timetable but be flexible and adaptable, which may change depending on patient/hospital priorities. |
| Interview 1 | Participants acknowledged that they did have skills despite lacking confidence. They also discussed the need for confidence to ask for help. | The placement model made them ready to go out on their own  “I think having done the long-arm placements, I was a lot more ready to just go out on my own.” Participant 6 Interview 1 | You need to be flexible and adapt to changing plans. Both within sessions and plans for the day. |
| Interview 2 | Participants discussed strategies to boost their confidence, such as “when not confident, calm myself down”  The independence of the placement model made them more confident in seeing clients on their own.  “My confidence has continued to build” Participant 1 Interview 2. “But a year on now I feel like I can do it from the back of my hand, it's just routine now in a way.” Participant 3, Interview 2 | “I am more confident seeing patients by myself and trusting their clinical reasoning/judgements. I am also able to work independently without consistently consulting people (senior clinician/supervisor).” Participant 4 Interview 2  Attributed working independently to work readiness. “Ability to work independently” Participant 2 Interview 2 | The ability to adapt and move away from home increased their ability to adapt and be flexible.  “The procedure is different at every facility, so I need to check with management and adapt as required. “Participant 2 Interview 2.  Being work ready is being flexible. “You have a generic plan, but you need to be flexible” Participant 5 Interview 2 |
